# Consuming sex: the association between modern goods, lifestyles and sexual behaviour among youth in Madagascar

**DOI:** 10.1186/1744-8603-9-13

**Published:** 2013-03-19

**Authors:** Kirsten Stoebenau, Rama C Nair, Valérie Rambeloson, Paul Ghislain Rakotoarison, Violette Razafintsalama, Ronald Labonté

**Affiliations:** 1 International Center for Research on Women, 1120 20th Street NW, Suite 500, Washington, DC, 20036, USA; 2 Vice Dean, Professional Affairs, Epidemiology and Community Medicine, Faculty of Medicine, University of Ottawa, 2129 P-451 Smyth Road, Ottawa, ON, K1H 8M5, Canada; 3 Statisticien Economiste, Lot II A 119 K Bis Soavaimbahoaka, Antananarivo, 101, Madagascar; 4 Department of Sociology and Anthropology, University of Antananarivo, Malagasy Socio-Consulting and Communication, Lot VS 52 QAL bis Avaratr’Ankatso, Antananarivo, 101, Madagascar; 5 Malagasy Socio-Consulting and Communication, Lot VS 52 QAL bis Avaratr’Ankatso, Antananarivo, 101, Madagascar; 6 Canada Research Chair, Globalization/Health Equity, Institute of Population Health, 1 Stewart Street, Ottawa, ON, K1N 6N5, Canada

**Keywords:** Transactional sex, Sexual behaviour, Madagascar, Modernity, HIV vulnerability, HIV risk, Globalization

## Abstract

**Background:**

Ethnographic evidence suggests that transactional sex is sometimes motivated by youth’s interest in the consumption of modern goods as much as it is in basic survival. There are very few quantitative studies that examine the association between young people’s interests in the consumption of modern goods and their sexual behaviour. We examined this association in two regions and four residence zones of Madagascar: urban, peri-urban and rural Antananarivo, and urban Antsiranana. We expected risky sexual behaviour would be associated with interests in consuming modern goods or lifestyles; urban residence; and socio-cultural characteristics.

**Methods:**

We administered a population-based survey to 2, 255 youth ages 15–24 in all four residence zones. Focus group discussions guided the survey instrument which assessed socio-demographic and economic characteristics, consumption of modern goods, preferred activities and sexual behaviour. Our outcomes measures included: multiple sexual partners in the last year (for men and women); and ever practicing transactional sex (for women).

**Results:**

Overall, 7.3% of women and 30.7% of men reported having had multiple partners in the last year; and 5.9% of women reported ever practicing transactional sex. Bivariate results suggested that for both men and women having multiple partners was associated with perceptions concerning the importance of fashion and a series of activities associated with modern lifestyles. A subset of lifestyle characteristics remained significant in multivariate models. For transactional sex bivariate results suggested perceptions around fashion, nightclub attendance, and getting to know a foreigner were key determinants; and all remained significant in multivariate analysis. We found peri-urban residence more associated with transactional sex than urban residence; and ethnic origin was the strongest predictor of both outcomes for women.

**Conclusions:**

While we found indication of an association between sexual behaviour and interest in modern goods, or modern lifestyles, such processes did not single-handedly explain risky sexual behaviour among youth; these behaviours were also shaped by culture and conditions of economic uncertainty. These determinants must all be accounted for when developing interventions to reduce risky transactional sex and vulnerability to HIV.

## Introduction

Ethnographic evidence suggests that transactional sex (sex provided in eventual exchange for gifts or money) is sometimes motivated by youth’s interest in the consumption of designer goods as much as it is in basic survival. This form of transactional sex has been described both as in step with “must-have” consumerist culture (e.g. [1,2]), and also as a means to an end in a world of increasing economic instability- women use their sexual prowess toward socio-economic mobility [3]. Here we assess with quantitative approaches the extent to which young women are, as one young man stated in a focus group in Antananarivo, Madagascar “laying down their bodies for the love of [fashion].” Is there any evidence to suggest that young women who report having either multiple sexual partners or having engaged in transactional sex are more interested in pursuing a “modern” lifestyle and obtaining the goods that symbolize it? More specifically, we examine whether or not there are associations between young people’s interests in the consumption of modern goods; their actual consumption patterns; and their sexual behaviour. In so doing, we contribute one of the first quantitative assessments of any association between consumption and sexual behaviour.

## Background

### Transactional sex and multiple sexual partners- linkages and definitions

Self reports of having had multiple or concurrent sexual partners in the last year [4-6], or informally exchanging sex for gifts or money (transactional sex) are identified as risk factors for sexually transmitted infections (STI) and HIV [7,8]. The term “transactional sex” has been adopted by public health and social science researchers to distinguish more formal, immediate sex-for-money exchange denoted as “prostitution” or “commercial sex work” from less formal forms of exchange embedded in relationships [9-12]. Specific additional designations are often used to characterize the exchange of sex for money or materials depending on the context, culture and circumstances- such as “survival sex,” [13], “cross-generational sex” [14], “sugar-daddies or mommies” or “sex for consumption” [15]. There are also important linkages between having multiple partners and the practice of transactional sex. Descriptions of women’s motivations to have multiple partners are often rooted in the potential for economic benefits from these partners [16-18]. Women also recount the implicit obligation to provide sex following any receipt of material or monetary benefit from a romantic partner [13,19]. As Kaufman and Stravou find among young people in Durban, South Africa, gift-giving among young people in romantic relationships with their peers is often associated with sex [20]. Finally, the sugar-daddy phenomenon (young woman having a transactional relationship with an older, and typically married man) almost always means concurrent sexual relationships for men [12,21] and can often imply concurrent relationships for the young women as well, who may pursue relationships with a peer in addition to their sugar-daddy [15,22].

#### A newer paradigm: exploring transactional sex motivated by “consumption”

The more conventional understanding of transactional sex and women’s heightened vulnerability to HIV is explained through women’s disproportionate economic disadvantage arising from gender discrimination. Women are described as passive victims with little choice but to resort to “survival sex,” exchanging sex for basic needs for themselves and their children [13,23,24]. This relationship has also been described in the context of economic globalization as macro-level economic crises have micro-level consequences that lead to increased likelihood that women have to rely on transactional sex [25,26].

More recent work has shed light on a different set of factors leading to transactional sex- less poor young women seek the stuff of “modernity” through engaging in economically-motivated sexual relationships. Young people, and their parents, in many different contexts across sSA describe a perception of “moral decay” that has accompanied the liberalization of economies, and an increased reliance on monetized capital accumulation [3,19,27,28]. Increasingly documented in ethnographic research is the notion that young people, especially women, are actively using their sexuality to obtain modern “designer” goods (i.e. cell phones, fashionable clothing) that are increasingly available but, for most, unaffordable. This perceived linkage between interests in consumption and sexual behaviour has been documented in urban and peri-urban contexts in sub-Saharan Africa; [1,2,12,17,29-32] and in Madagascar in particular [3,18,33]. Very few studies examining transactional sex in the rural context draw from this paradigm (but see [28,34]). While evidence is mounting in the ethnographic record, the extent to which interest in the consumption of modern “designer” goods motivates risky sex has yet to be examined quantitatively. A few studies do however provide some understanding about the details of the transaction. In a study of women in Soweto, South Africa, Dunkle (2004) [7] quantitatively assessed what goods women who reported having had transactional sex received. Luke [29,35,36] examined the amount of the “transfer” offered by men in their informal exchange relationships with women and found that higher transfers are associated with riskier sexual behaviour (namely forgoing the use of a condom).

More recent reviews of the literature have suggested that there may not be such a clear dichotomy between the survival sex of the passive victim and the consumption sex of the active sexual agent, rather, these distinctions are far more nuanced [22,37]. Kuate-Defo coined the phrase “economic vulnerability” to capture both relationships motivated by “survival” as well as conformity, fashion, social status and pride among peer group members [15]. Another rendering presented by Jennifer Cole described young women on the east coast town of Tamatave, Madagascar who actively pursued relationships with foreign men through accessing the “right” clothes and accessories, both toward social and economic advancement as well as toward the hopeful eventuality of finding a foreign spouse [3,38] (see also [39] for similar findings among university students in Zimbabwe).

We drew from two contrasting sites in Madagascar to examine the association between interests in consuming modern goods and sexual behaviour, and the extent to which regional differences play a role. We examine these associations in the large national capital city of Antananarivo and its peri-urban and rural surroundings, and the northern port town of Antsiranana.^a^ While households in the compared urban areas report similar expenditure levels and close to similar average educational attainment, the employment sectors differ, as do socio-cultural profiles.

### Sites of transformation: Antananarivo and Antsiranana Madagascar

#### Transforming socio-economic contexts

More recent political and economic transformations in Madagascar increase the likelihood that women and men have become more “economically vulnerable,” and therefore that women may have had to rely on their sexuality to buffer uncertainty. In the last 10 years Madagascar has endured two political crises, the latter of which began in 2009 and is ongoing. In the last thirty years Madagascar has transitioned from isolationist Marxist-Socialism to globally-integrated neoliberalism [3,40]. The relatively late and then rapid insertion into the global economy catalyzed a number of changes. Most significant and relevant perhaps are the introduction of export processing zones (EPZs), the relaxation on import tariffs, the increases in the tourist sector, and the increasing ineffectiveness of tertiary education [41-47]. These different aspects of the transforming economic reality in Madagascar affected the two study sites similarly in some cases and differently in others. Antananarivo, located in the center of the country, is arguably the most “global” city in Madagascar. The capital city of nearly 2 million inhabitants, it was most impacted by the expanding EPZ sector, as well as the influx of imported goods and specialty stores. Antananarivo now hosts numerous internet cafes, and has seen a dramatic rise in shopping centers and boutiques, restaurants and bars in the last 20 years. These service points cater not only to foreign tourists and business people, but more recently to a small population of wealthy Malagasy. Antsiranana is about one tenth the population of Antananarivo and is located on the northern coast, built at the bottom of a large, U-shaped bay and boasting a large, functioning port; the second largest in Madagascar, it is a destination for naval and cargo ships, as well as tourists en route to its nearby beaches and national parks.

Foreign-direct investment resulted in increased possibilities for low skilled workers in Madagascar. Madagascar experienced near unmatched increases in the export-processing zone (EPZ) sector as compared to the rest of SSA through the late 1990s (20% annual increase between 1997–2001) [42]. EPZs are almost exclusively located in urban or peri-urban Antananarivo and young women make up most of the workforce [41].

Alongside export-led growth strategies, loan conditionalities of the IMF and World Bank and trade agreements have led to reductions in import tariffs, and subsequently, imports continue to outpace exports [43,44].^b^ While data are difficult to locate for import trends for designer clothing and boutiques in particular, technology imports implied by the rate of change in cell phone subscriptions provide some indication. Not unlike the trend in other sub-Saharan African countries, there has been an exponential increase in cell phone subscriptions over the last two decades. While 2 in 1000 individuals had a cell phone in 1994, this was up to 30 in 1000 by 2005, and had increased to a remarkable 300 in 1000 by 2009 [45].

Another significant “import” are tourists, who have been coming to Madagascar at a rate of increase of about 20,000 tourists per year since the early 1990s. (ibid) Following recovery from a crippling but short-lived political crisis in 2002, the rate of increase jumped to 30,000 or 40,000 additional tourists per year [45]. The investment in the tourist industry in Antsiranana has soared in the last 15 years: between 2004 and 2008 alone, the estimated annual number of tourists to the city and surrounding region nearly doubled from about 15,000 to almost 27,000; particularly significant for a city of only about 200,000 [46].

There are some worrying indicators with regard to social and economic mobility in Madagascar. Reliance on foreign direct investment has been accompanied by gains in employment opportunities for low-skilled jobs, as major corporations have tended to import more technically skilled workers. In addition, emphasis on privatization has reduced public sector and stable employment possibilities. Unemployment among 25 year olds in the capital city of Antananarivo in the late 1990s was almost twice that experienced by the previous generation. One researcher commented that in SSA cities such as Antananarivo, a university diploma “has become a risk factor for finding employment” [47]. Evidence from the World Bank shows that from 1993 to 2005 change in the share of total unemployment for those with tertiary education more than doubled from 4.1% to 9.3%; and was more severe for men than for women. During the same time period, the share of total unemployment for those with a secondary education declined significantly from 37% to 24% [45]. While those with a tertiary level education comprise only a small percentage of the total population (about 3%), these trends carry significance for understanding the pathways to economic and social mobility for society as a whole.

#### Transformations and distinctions in sexual behaviour

Regional distinctions in family formation and sexual and reproductive behaviour in Madagascar have been noted in the ethnographic record and can be deduced from more recent demographic data collection [3,48-52]. (It is important to note here that administrative divisions in Madagascar officially changed from 6 provinces to a further decentralized 22 regions (*faritra*) in 2007- both the cities of Antananarivo and Antsiranana were the urban centers of provinces of the same name until 2007. We refer below to these older administrative divisions.) For example, Demographic and Health Survey data from 2004 show that among 25–29 year old women the weighted median age at first sex in the province of Antsiranana was 15.7, while it was 18.7 in the province of Antananarivo. Age at first union (either formal marriage or cohabitation) is correspondingly much younger in Antsiranana than Antananarivo (in 2004 median age at first union was 18.2 in Antsiranana, and 20.7 in Antananarivo) [53]. This distinction may be explained in part by a 2 year gap, as of 2004, in the mean level of educational attainment for 25–29 year olds in Antananarivo as compared to Antsiranana at the provincial level (as opposed to within the respective cities, where the education gap has closed) [54,55].

There are also differences in the reporting of multiple sexual partners (2 or more sexual partners in the last 12 months) across these regions. According to Youth Sexual Behaviour survey data, among young never married women (ages 15–24) in Antananarivo, the percent reporting having had multiple partners in the last year rose from 5% in 2004 to 9% in 2008; in Antsiranana the corresponding figures moved from 7% to 23% [56-58]. Among men, the pattern is similar, but the magnitude is higher in both areas. These regional distinctions in reported sexual behaviour are not totally unexpected. Ethnographers have documented the relative openness and acceptance of premarital sexuality within certain ethnic groups in Madagascar (for example see: [3,52,59]). The area including and surrounding Antananarivo is primarily inhabited by those who self-identify with the Merina ethnic group. The Merina were more highly exposed to and accepting of European missionary efforts beginning in the early 1800’s than many other groups in Madagascar. Urban elite Merina in the city of Antananarivo identify as Christian and contrast their (self-described) chastity with the sexual behaviour of other ethnic groups and social classes; explaining this distinction in large part through Christian ethos, and using it as a basis for ethnic-related discrimination [60]. Many Merina sociologists would suggest that these distinctions are exaggerated, and that Merina are simply less inclined to disclose their sexual practices than other ethnic groups ([61,62]. It is the case, however, that not only reports of risky sexual practices, but also HIV prevalence rates, are among the lowest in Madagascar within the Antananarivo province (it is also the case that infrastructure, education levels, and access to healthcare are better in this region as compared to others) [63,64].

National surveillance studies of unmarried youth beginning in 2004 asked about engagement in transactional sex, wording the question as follows: “Have you ever received gifts or money in exchange for sex?” In many parts of Madagascar, this exchange is implicit among a normative set of prescriptions in sexual relationships. Therefore, the observation that there are differences between the provinces of Antananarivo and Antsiranana in the prevalence of reporting of engagement in transactional sex should come as no surprise. In both Antananarivo and Antsiranana the percentage of women who report having received money or material items in exchange for sex rose between 2004 and 2008; in Antananarivo from 1.2% to 5.6%; in Antsiranana, from 22% to 57% [56-58]. In parts of northern and eastern Madagascar a woman was traditionally expected to receive remuneration as a token of respect following sexual relations [52,59]. Therefore, the meaning of this finding from these surveillance studies is ambiguous. It is difficult to know how these questions are understood by the respondents, and a reported rise in this behaviour may just refer to a rise in sexual activity among this population group- a finding worth consideration, but not the intended meaning of the question. In addition, no study has recorded sexual behaviour alongside ethnic identity (governmental-funded surveys have not been permitted to include questions regarding ethnicity since the early 1970s), place of residence and socio-economic indicators. In this study we are better able to differentiate the influence of ethnic identity from other known influences on sexual behaviour.

#### Research questions and study expectations

The increasing evidence of the linkages between transactional sex or multiple sexual partners and HIV has led to calls for greater investment in prevention efforts that try to address these behaviours, particularly among youth [65,66]. Work on understanding the determinants and associations with these behaviours, from the structural to individual level, is therefore imperative for HIV and STI prevention efforts. We contributed to this area of inquiry by asking: *Is there an association between young people’s consumption of “modern” goods and reporting of risky sexual behaviours?* Specifically, we examined linkages with the reporting of multiple partners in the last twelve months or having ever engaged in transactional sex*.*

Research describing sex for consumption has often been based in urban contexts. Certainly access and exposure to the stuff of modernity are more apparent in urban landscapes. Limited research has also found independent effects of the urban environment on risky sexual behaviour [67]. This relationship has been explained by both the anonymity, and often the greater diversity (ethnic, religious, linguistic) of urban centers, which together may reduce the effectiveness of communal-based social sanctioning systems [67,68]. We explored the linkages between consumption and sexual behaviour across type of residence *expecting to find a higher prevalence of reported riskier behaviour, levels of consumption, and expressed interest in consumption in urban areas*.

Finally, *we expected to find strong socio-cultural associations with sexual behaviour, particularly for women*. Distinctions in belief systems and practices regarding sexual permissiveness and the meaning of monetary exchange within sexual relationships differ between the cultural groups inhabiting the regions of Antananrivo and Antsiranana. Specific cultural belief systems have been described together with increasing reliance on market economies as important influences on patterns of transactional sex in various parts of sub-Saharan Africa ([69] in Lesotho; [52] in Madagascar; [70] in Kenya;[2] in South Africa).

In order to address the relationship between consumption and sexual behaviour, we first determined the meaning of modern goods and a modern lifestyle in the study sites of interest through formative qualitative research. We then measured the consumption patterns and reported sexual behaviours of youth in the urban, peri-urban and rural context and in two regions. Below we detail the methodological and analytical approach.

## Methods

This paper is based primarily on quantitative data collection in Antananarivo and Antsiranana, Madagascar in 2009. Formative focus groups (n = 34; 8 with parents; 26 with youth ages 15–24) directed the population-based survey of youth in these two regions. This study was reviewed and approved by both the National Ethical Review Board of Madagascar and the Ottawa Hospital Research Ethics Board (OHREB).

### Sampling frame and data collection: population-based survey of youth, Antananarivo and Antsiranana

The population-based survey of youth ages 15–24 was drawn equally from the urban, peri-urban and rural residence zones of Antananarivo in order to examine the influence of zone of residence on sexual behaviour. In order to draw a comparison of the regional socio-cultural effects on sexual behaviour, we include urban Antsiranana as a comparison to urban Antananarivo. We used a three-tiered sampling procedure, randomly selecting census blocks; households and finally individuals within households. In total, 25 census blocks were selected in Antananarivo and 10 in Antsiranana with probability proportionate to their size. Households were randomly selected based on a sampling interval calculated from the total number of eligible households in the census block and the necessary number of young men and women to be included in the survey from each census block. Using a map of the census block obtained from local authorities and a pre-defined route, households were randomly selected at a pre-determined interval until the necessary number of households with eligible participants was identified. Only one eligible respondent was interviewed from any selected household in order to avoid interviewing husbands and wives in the same household given the sensitive nature of the questionnaire, as well as to increase the dispersion of the sample within each census block. We did not exclude respondents based on marital status as men, in particular, are assumed to continue to pursue additional sexual partners regardless of their marital status in every region of Madagascar.

Based on findings from 2004 and 2006 data on reported sex for money exchange among youth in urban as compared to rural Antananarivo we estimated the necessary sample size to detect a difference of 5% on reported sex for money exchange among youth at a statistical significance level of less than 5%, and statistical power of 80%. Our sample in Antsiranana was calculated to capture about 5% of the estimated total population of sexually active women ages 15–24 reporting sex for money exchange in urban Antsiranana. We also accounted for the percentage of youth who are not yet sexually active (~40% of females and ~36% of males), and assumed a survey participation refusal rate of 6%. The sample size for the population-based survey by study site is provided in Table 1 below.

**Table 1:** Population-based survey sample size by study site, Madagascar, 2009-10

	**Antananarivo**	**Antsiranana**	**Total**
Type of residence			
Urban	625	380	1005
Peri-Urban	625	-	625
Rural	625	-	625
Total	1875	380	2255

Fieldwork and data collection took place between September, 2009 and February, 2010. For the focus groups, we purposively selected a small sub-set of communities from the universe of those randomly selected for inclusion in the quantitative survey. Focus groups were conducted prior to quantitative data collection and initial findings were used to build the study instrument (see [19] for more detailed results from these focus groups).

The survey took from 30 minutes to one hour to complete. It was comprised of six main sections: Household characteristics; socio-demographics of the respondent; detailed description of respondents’ last month’s income and expenditures; respondent’s preferences and interests- e.g. type and frequency of engagement in leisure activities; perceptions around the importance of keeping up with fashion; and respondent’s sexual behaviour, including characteristics of recent sexual partners.

### Analytic methods

Below we describe the variables explored in our analyses including our outcome, explanatory and control variables and then summarize the steps we took in our analysis.

#### Sexual behaviour outcomes

We modeled ‘reporting having had multiple sexual partners in the last year’ as well as ‘reporting having exchanged sex in order to receive gifts or money in one’s lifetime’. Respondents reporting having had two or more sexual partners in the last year were considered as having had multiple partners. We focused on what distinguishes those who reported only having had one partner from those who reported having had multiple partners.

We carefully worded the question designed to assess whether or not respondents had engaged in transactional sex in their lifetime given the normative understanding of exchange within sexual relationships. We stated the motivation behind the exchange asking “Have you ever had sex *with the intention *of receiving gifts or money in return?”^c^ We expected this wording would result in a lower percentage of respondents answering in the affirmative as compared to other surveys, particularly in Antsiranana.

#### Explanatory variables

Explanatory variables included “modern” goods and lifestyle characteristics and perceptions of their importance; region and type of residence; and ethnic identity.

##### Modern goods and lifestyles

The focus group discussions explored connotations of *lamaody* (from “la mode”, translates to both ‘fashion’ and ‘leading a fashionable lifestyle’), and found that foremost, it referred to fashionable clothing and goods, including technology. In addition, focus groups revealed a semantic association between sexual behaviour and a fashionable lifestyle- participants’ described both early sexual debut and having multiple sexual partners as becoming *lamaody*, for both young men and women. Finally, it was considered *lamaody* to “mi-reve” or “to party,” including drinking alcohol, and going out to nightclubs and karaoke establishments.

Based on these findings, we measured fashionable goods and lifestyle as follows:

1. Clothing: We built response categories for a quantitative count of the number of *lamaody* clothing items (e.g. skinny jeans; ballerina shoes; timberland boots) respondents’ had purchased, received, or “found” (euphemism for stolen) in the last six months. We collapsed this count to a categorical variable: 0 items; 1 to 3 items; or more than four items.

2. Technology: We included whether or not the respondent had made a call on their cell phone the previous day (as cell phone ownership does not necessarily translate into regular usage) and if they had used the internet in the last six months.

3. Modern lifestyle: We assessed two activities that were associated with modern youth lifestyles- alcohol consumption in the last four weeks and having gone to a nightclub in the last six months. Alcohol consumption tends to be strongly associated with riskier sexual behaviours [8,13,71]. Alcohol consumption is particularly characteristic of a “modern lifestyle” for women, as it has been traditionally considered inappropriate for women to consume alcohol.

We also measured whether or not the respondent had communicated with a foreigner from outside of Madagascar in the last six months. The wording of this question was intended to capture young women (less often men) interested in trying to establish romantic relationships with foreigners. Because some of these women rely on the internet to establish communication, we were careful to word the question so that it did not necessitate an in-person meeting.

Two questions were used to gauge respondents’ perceptions concerning *lamaody*. 1. Respondents’ were asked how important it is to follow *lamaody*. We differentiated between three categorizations: not at all important; somewhat important; or important. 2. Respondents’ were also asked if they consider themselves to be someone who follows *lamaody*. Six possible response categories varied from never to always. We collapsed these to “rarely or never,” “sometimes,” and “for parties, often, or always.”

##### Type of residence

We included respondents’ region and type of residence through a categorical variable that differentiated between urban, peri-urban and rural Antananarivo, and urban Antsiranana.

##### Ethnic identity

It is considered taboo to ask someone their ethnic identity, therefore we asked for the location of the respondent’s father’s tomb, which represents their patrilineal ethnic ties. We included ethnic origin as a dichotomous variable, and differentiated those who identify as having originated from regions that are primarily inhabited by the Merina (which is foremost the region surrounding and including urban Antananarivo, historically known as *Imerina*); from those who did not identify as having originated from *Imerina*. We collapsed these codes as such due to the small overall sample of non-Merina in these data.^d^

#### Control variables

The literature points to important socio-demographic and socio-economic determinants of sexual behaviour for young men and women that we captured in our analyses using the following variables: age, education, household asset-based wealth, last month’s expenditures, whether or not the respondent received money from their parents, and household composition.

Education has been associated with lower levels of sexual behaviour, such as delayed initiation of sexual behaviour, or lower rates of multiple partners [72]. We use highest level of education attained in single years (range 0–17).

Research is somewhat equivocal on the relationship between socio-economic status and likelihood of reporting multiple sexual partners or transactional sex. A number of studies support the expected gender dynamic wherein lower economic status is associated with risky behaviour for women [73-77]; while relatively higher socio-economic status is associated with risky sexual behaviours for men [21,35,78,79]. We used three variables to assess respondent’s background socio-economic status as well as disposable income: household asset based wealth, respondent’s last month expenditures, and whether or not a respondent reported their parents as a source of their personal income (as a binary variable). For the latter, the respondent was asked “What all do you do to earn money?” and “pocket money from parents” was among the response categories. We included this variable as it represents respondent’s personal expenditure capability independent of monies they might receive from a sexual partner; it is associated with household wellbeing; and it may also indicate parental attitudes about the extent to which young women or men should be supported as opposed to contribute to the household during this life stage. Research has suggested that parents may allow or subtly encourage girls’ to seek boyfriends who will support the girl [34].

Household-based asset wealth was constructed from a multiple correspondence analysis (MCA) of 19 variables describing characteristics of the home and the household’s major assets.^e^ Results from MCA have been found equivalent to principle components (PCA) based asset-indices; but MCA is better suited to categorical variables [80]. The household wealth index score was divided into quintiles and then further reduced to a binary variable distinguishing the two poorest quintiles from the three non-poor quintiles.

We collected last month’s income and expenditure data from *our individual youth respondents*, not their households. We rely on expenditures, as these data are known to estimate income from all sources more accurately than reported income. Last month’s total expenditures is included as a categorical variable with five categories based on the sample distribution ranging from less than 18,000 Malagasy Ariary (about 9 USD) to more than 100,000 Malagasy ariary (about 50 USD). We use this variable only for modelling men’s sexual behaviour as women may be reporting money they have received from sexual partners (29.6% of young women reported their partner as a source of income as compared to only 0.7% of men).

Finally, we include respondent’s household composition, as among never married youth, living with both parents has been found to have a significant impact on sexual behaviour in some parts of sSA [81]; and in Madagascar sociologists have suggested lack of parental oversight as a determinant of risky adolescent sexual behaviour [33,50]. Following exploratory analysis that showed no statistically significant difference in outcomes for those living with both versus either parent, we operationalized this variable in to three categories: respondent lives with either or both parents; respondent lives with spouse or partner; or respondent lives with others (family or non-family members).

#### Analysis plan

Data were double entered into Epi-Data and transferred into Stata (Version 10) and SPSS (Version 14) and after cleaning and re-coding data, a series of descriptive statistics were obtained to assess distributions of outcomes, controls and explanatory variables of interest. We present all of our findings stratified by sex, as the prevalence and determinants of sexual behaviour are known to differ by sex. Gendered expectations for sexual behaviour often result in a sexual permissiveness granted to men that is not extended to women [78,82,83]. Thus, in most settings men are more likely than women to report having had multiple or multiple and concurrent partners ([78,84]) and given persistent gendered-power structures, women are more likely than men to report having received goods or money in exchange for sex (but see [79,85]).

We present below descriptive statistics of the study population stratified by sex and type of residence and region (Tables 2 and 3). We then explore associations with our explanatory variables in greater detail with descriptive and bivariate statistics (Tables 4 and 5). Lastly, we present multivariate logistic regression models examining our outcomes (Table 6). To build these models, we first examined bivariate statistics with explanatory and control variables. In addition, we tested the strength of the association between our outcomes and each of our explanatory variables in models that adjusted for socio-economic and socio-demographic controls, specifically: age, education, household composition, household asset-based wealth, financial support from parents, and for men only, personal expenditures. We used the results from these analyses to build full models that test the relative strength of the associations of ethnic identity, type of residence and consumption and lifestyle with sexual behaviour when controlling for socio-economics and socio-demographics. We also tested these models for multi-collinearity and goodness-of-fit (using the Hosmer-Lemeshow goodness of fit test).

**Table 2:** Selected characteristics for young men (ages 15–24) by region and type of residence, Madagascar (2009)

	**Antananarivo**			**Antsiranana**
	**Rural (n = 324)**	**Peri-Urban (n = 325)**	**Urban (n = 326)**	**Urban (n = 200)**
***Socio-demographic characteristics***				
Mean Age [SD]	18.7 [2.68]	18.5 [2.72]	18.9 [2.69]	18.5 [2.70]
In union (legally married or common law) (%)	9.9	7.4	8.9	6.5
Household composition (%)				
Lives with at least one parent	78.4	80.3	70.3	57.5
Lives with spouse/partner	8.3	7.4	11.0	9.0
Lives with other family/non-family	13.3	12.3	18.7	33.5
Region of origin- *Imerina* (%)	97.2	86.2	84.1	10.5
***Socio-economic characteristics***				
Mean highest level of Education [SD]	6.8 [2.70]	7.8 [2.95]	9.0 [3.06]	9.0 [2.57]
Median Expenditures last mth (in 1000 MGA) [IQR]	24 [4, 60]	15 [4, 45]	20 [6.8, 60]	10 [4, 30]
Receives money from parents (%)	16.1	49.3	64.9	75.5
Household in 2 poorest quintiles (%)	86.1	26.5	21.8	10.0
***sexual behaviour characteristics***				
Have ever had sex (%)	43.8	46.5	58.3	61.5
Had two or more sex partners in last 12 months (%)	32.2	23.1	32.5	34.8
***Consumption of designer goods and modern lifestyle***				
Used their cell phone yesterday (%)	9.6	13.9	21.5	32.0
Fashionable items obtained in last six months (%)				
0 items	39.5	24.3	9.5	39.0
1-3 items	51.9	45.2	43.6	37.0
4 + items	8.6	30.5	46.9	24.0
Used internet in last six months (%)	2.8	6.8	30.4	19.0
Went to nightclub in last six months (%)	4.9	7.7	12.0	18.5
Drank alcohol in last 4 weeks (%)	39.5	21.2	23.0	28.0
*Perceptions concerning fashion*				
I am fashionable (%)*:*				
Rarely/Never	87.7	80.6	97.9	80.0
Sometimes	6.2	13.5	1.5	7.5
For Parties/Often/Always	6.2	5.9	0.6	12.5
Fashion is important (%):				
Not at all	64.5	44.9	38.7	55.0
Somewhat	31.2	47.1	54.9	36.0
Important/Very Important	4.3	8.0	6.4	9.0

**Table 3:** Selected characteristics for young women (ages 15–24) by region and type of residence, Madagascar (2009)

	**Antananarivo**			**Antsiranana**
	**Rural (n = 299)**	**Peri-Urban (n = 300)**	**Urban (n = 299)**	**Urban (n = 180)**
***Socio-Demographic characteristics***				
Mean Age [SD]	18.2 [2.66]	18.5 [2.69]	18.8 [2.75]	18.6 [2.67]
In union (legally married or common law) (%)	28.2	24.0	18.1	15.6
Household composition (%)				
Lives with at least one parent	63.8	61.7	58.9	41.7
Lives with spouse/partner	25.9	23.7	17.7	18.3
Lives with other family/non-family	10.3	14.7	23.4	40.0
Region of origin- *Imerina* (%)	93.4	88.0	78.6	6.7
***Socio-economic characteristics***				
Mean highest level of Education [SD]	6.7 [2.71]	7.6 [3.25]	8.6 [3.30]	8.3 [2.96]
Median Expenditures last mth (in 1000 MGA) [IQR]	18 [2, 60]	20 [4, 78]	20 [5.6, 48]	21.5 [9.3, 81]
Receives money from parents (%)	30.7	43.8	42.9	41.7
Household in 2 poorest quintiles (%)	81.4	34.7	24.8	12.8
***sexual behaviour characteristics***				
Have ever had sex (%)	42.9	47.3	41.8	61.7
Had two or more sex partners in last 12 months (%)	1.8	3.9	5.5	19.4
Have ever had sex in order to receive goods/money (%)	0.0	4.9	0.80	19.8
***Consumption of designer goods and modern lifestyle***				
Used their cell phone yesterday (%)	12.6	15.3	25.1	33.9
Fashionable items obtained in last six months (%)				
0 items	21.6	24.3	22.7	35.6
1-3 items	54.5	43.3	42.8	36.1
4 + items	23.9	32.3	34.5	28.3
Used internet in last six months (%)	2.3	7.0	19.4	11.7
Went to nightclub in last six months (%)	0.7	2.3	5.4	8.3
Drank alcohol in last 4 weeks (%)	5.6	3.3	7.7	1.7
*Perceptions concerning fashion*				
I am fashionable (%)*:*				
Rarely/Never	74.8	75.3	82.9	73.9
Sometimes	13.3	5.0	14.4	19.4
For parties/Often/Always	12.0	19.7	2.7	6.7
Fashion is important(%):				
Not at all	27.9	59.3	55.5	55.0
Somewhat	55.8	38.0	32.1	39.4
Important/Very Important	16.3	2.7	12.4	5.6

**Table 4:** Association between consumption and sexual behaviour for young men and women (ages 15–24), Antananarivo and Antsiranana, Madagascar, 2009

	**% Reporting two or more sexual partners in last 12 months**						**% Ever exchanged sex in order to receive gifts/money**		
	**Men**			**Women**			**Women**		
	**Yes**		**No**	**Yes**		**No**	**Yes**		**No**
	**30.7**		**60.9**	**7.3**		**92.7**	**5.9**		**94.1**
	**(n = 154)**		**(n = 347)**	**(n = 33)**		**(n = 421)**	**(n = 30)**		**(n = 477)**
*perceptions concerning fashion*									
I am fashionable*:*									
Rarely/Never	79.2		85.3	51.5		75.8	53.3		75.3
Sometimes	12.3	^+^	7.8	**30.3**	***	13.1	23.3	^+^	14.1
For Parties/Often/Always	8.4		6.9	**18.2**	^+^	11.2	**23.3**	**	10.7
Fashion is important:									
Not at all	37.1		45.0	27.3		48.7	33.3		49.1
Somewhat	48.7		47.6	**60.6**	**	41.8	56.7	^+^	41.5
Important/Very Important	**14.3**	**	7.5	12.1		9.5	10.0		9.4
Cell phone use yesterday	**34.4**	**	23.3	**48.5**	***	22.8	36.7	^+^	21.6
Drank alcohol in last 4 weeks	**50.0**	***	35.7	**21.2**	***	5.5	10.0		6.5
Fashionable items obtained in last six months:									
0	19.5		29.4	24.3		29.5	40.0		29.2
1 to 3 items	39.6		40.6	24.3		41.1	**20.0**	**	40.9
More than 4 items	**40.9**	***	30.0	51.5		29.5	40.0		30.0
*In the last six months:*									
Used the internet	**22.7**	**	13.8	**27.3**	***	6.7	13.3		7.6
Went to a nightclub	**28.6**	***	12.4	**30.3**	***	4.3	**23.3**	***	4.6
Got to know a foreigner	1.3		0.3	**24.3**	***	1.9	**20.0**	***	2.1

*Indicates statistical significance for those categories or variables that predict sexual behaviour outcomes in bivariate logistic regression models.

^+^p < .1 **p < .05 ***p < .01.

**Table 5:** Sexual behaviour by ethnic origin and current residence among young men and women (ages 15–24) in Antananarivo and Antsiranana, Madagascar, 2009

	**Percent reporting 2+ sexual partners in last 12 months**		**Percent ever exchanged sex in order to receive gifts/money**
	**Men**	**Women**	**Women**
**Ethnic origin- overall**			
*Imerina* origin	28.5	2.0	1.8
	(n = 347)	(n = 303)	(n = 335)
Other origin	35.7	17.9	14.0
	(n = 154)	(n = 151)	(n = 172)
**Ethnic origin- Urban Antananarivo**			
*Imerina* origin	30.2	0.0	0.0
	(n = 126)	(n = 80)	(n = 88)
Other origin	41.9	20.7	2.7
	(n = 31)	(n = 29)	(n = 37)
**Ethnic origin- Peri-Urban Antananarivo**			
*Imerina* origin	20.6	2.8	5.0
	(n = 97)	(n = 109)	(n = 119)
Other origin	35%	10.5	4.4
	(n = 20)	(n = 19)	(n = 23)
**Ethnic origin- Rural Antananarivo**			
*Imerina* origin	33.0	1.9	0.0
	(n = 112)	(n = 108)	(n = 122)
**Ethnic origin - Urban Antsiranana**			
Other origin	35.0	19.6	21.0
	(n = 100)	(n = 97)	(n = 97)

Note: “n” reflects denominator.

**Table 6:** Multivariate logistic regression models examining the odds of reporting having had multiple partners in the last 12 months or having ever engaged in transactional sex for young men and women (ages 15–24) in Antananarivo and Antsiranana, Madagascar (2009)

	**% Reporting two or more sexual partners in the last 12 months**				**% Ever had sex intending to receive gifts/money in return**	
	**Men (n = 501)**		**Women (n = 454)**		**Women (n = 507)**	
	**OR**	**95% CI**	**OR**	**95% CI**	**OR**	**95% CI**
*Type of residence* (Ref = Urban)						
Peri-Urban	0.518**	(0.297 – 0.904)	1.014	(0.245 – 4.197)	–	
Rural	1.036	(0.544 – 1.970)	2.179	(0.303 – 15.68)	–	
Region of origin (Ref = *Imerina*)	–		7.211***	(2.109 – 24.66)	11.87***	(3.735 – 37.74)
*Perceives they are fashionable* (Ref = Rarely/Never)						
Sometimes	–		2.665	(0.678 – 10.48)	6.476**	(1.559 – 26.90)
For parties/Often/Always	–		1.295	(0.308 – 5.449)	2.436	(0.572 – 10.37)
*Perceives fashion is important* (Ref = Not at all):						
Somewhat	1.176	(0.754 – 1.833)	1.761	(0.556 – 5.582)	1.894	(0.578 – 6.209)
Important/Very Important	1.670	(0.822 – 3.393)	0.503	(0.065 – 3.877)	0.273	(0.0306 – 2.432)
Used their cell phone yesterday	–		1.556	(0.568 – 4.263)	1.684	(0.522 – 5.436)
Consumed alcohol in last 4 weeks	**1.485**^**+**^	(0.969 – 2.273)	5.261**	(1.282 – 21.59)	–	
*In the last six months:*						
Used the Internet	–		4.910**	(1.095 – 22.01)	–	
Frequented nightclub	2.201***	(1.296 – 3.736)	5.296***	(1.496 – 18.75)	10.02***	(1.779 – 56.41)
Got to know a foreigner	–		5.453**	(1.235 – 24.07)	14.53***	(1.955 – 108.0)
*Fashionable items consumed in last 6 months* (ref = 0)						
1–3	–		–		**0.325**^**+**^	(0.0883 – 1.197)
4 or more	–		–		0.659	(0.176 – 2.460)
Age	1.022	(0.928 – 1.126)	0.773**	(0.624 – 0.957)	0.983	(0.804 – 1.202)
Highest Education Attained	0.968	(0.896 – 1.046)	0.787***	(0.663 – 0.934)	0.761***	(0.629 – 0.919)
*Household composition* (Ref = Lives with at least one parent)						
Lives with spouse	**0.584+**	(0.314 – 1.087)	1.357	(0.332 – 5.538)	0.463	(0.140 – 1.529)
Lives with other family/non-family	0.928	(0.531 – 1.622)	1.486	(0.415 – 5.324)	0.339	(0.0853 – 1.345)
*Household Wealth* (Ref = 2 poorest quintiles)	1.329	(0.740 – 2.386)	6.987**	(1.392 – 35.06)	1.652	(0.507 – 5.383)
Receives pocket money from Parents	0.933	(0.524 – 1.661)	0.478	(0.140 – 1.631)	0.00787***	(0.000518 – 0.120)
*Last month’s expenditures* (in ariary) (Ref = <18,000)						
18,000–49,000	**1.663+**	(0.949 – 2.915)	–		–	
50,000–99,000	1.324	(0.716 – 2.446)	–		–	
> = 100,000	3.105***	(1.489 – 6.475)	–		–	

*** p < 0.01, ** p < 0.05, ^+^ p < 0.1.

## Results

### Describing the study population

#### Comparing contextual factors between urban Antananarivo and Antsiranana

Tables 2 and 3 describe the male (n = 1175) and female (n = 1080) study populations, respectively, by region and place of residence. These tables indicate that the urban populations of Antananarivo and Antsiranana were comparable for both sexes in terms of level of education, and age. While the likelihood of reporting living with a spouse or partner was about the same across cities for both sexes, it was more common that respondents reported living with at least one parent in Antananarivo as compared to Antsiranana, where respondents were more likely to report living with extended family members. Although respondents were less likely to live with their parents in Antsiranana, they were as likely (for women), or more likely (for men) to receive financial support from their parents. Last month’s median expenditures differed between urban men from Antananarivo (20,000 Malagasy Ariary; ~10 USD) as compared to Antsiranana (10,000 Malagasy Ariary, ~5 USD). This finding suggests that men in Antsiranana had less spending power than in Antananarivo, consistent with the finding also shown in Table 2, that these men additionally reported having purchased fewer modern goods. That said, households in urban Antsiranana tended to be wealthier than households in urban Antananarivo, overall.

For modern goods and lifestyle (*lamaody*) characteristics, socio-cultural or structural factors help explain some of the differences found across urban sites. Going to a nightclub is considered more socially acceptable in Antsiranana. In both locations, nightclubs are considered less appropriate for women to attend as compared to men, hence lower percentages of women reported having frequented a nightclub. Internet use was also considered *lamaody*- the internet was much harder to access in Antsiranana, where there were, as of 2009, very few functioning internet cafes; as compared to multiple such sites in Antananarivo. Finally, for alcohol consumption, given a significant percentage of the respondents in Antsiranana were Muslim (31% of men and 18% of women), one would expect lower reported levels of alcohol consumption in Antsiranana as compared to Antananarivo- this was the case for women, but not for men. The reported differences by region concerning perceptions around fashion did not provide a clear pattern; while women and men in Antsiranana were both more likely to suggest they were often fashionable; women in Antananarivo were more likely to suggest that fashion was very important (12.4% versus 5.6%).

#### Comparing contextual factors across residence type in Antananarivo

The socio-economic and demographic distinctions by place of residence (rural, peri-urban and urban) within Antananarivo often followed expected trends, particularly among women. Education levels rose from rural to urban place of residence for both sexes; and for women the percentage who reported they lived with a spouse or partner fell from rural to urban residence. As expected, given women tend to marry younger than men, a greater percentage of women reported residing with a spouse or partner, regardless of place of residence. For both men and women it was most common to report living with extended or non-family members in urban residence areas. Monthly expenditures were highest overall among rural men at 24,000 Ariary (about 12.00 USD), reflecting the greater likelihood that these men were heads of households and the primary household income earner. Finally, the percentage of households in the bottom two quintiles of the asset-based wealth index was much higher in rural areas as compared to peri-urban or urban areas.

The same trends from rural to urban were found for some modern goods and lifestyle characteristics. For both men and women, cell phone use, internet use, and having frequented a nightclub all rose from rural to urban place of residence. This was not the case, however, for reported alcohol consumption, where it was high in the rural areas, particularly for men, where 39% reported having consumed alcohol in the last four weeks, a difference of more than 11% from the next highest residence category. While expenditures on fashionable or modern goods rose from rural to urban place of residence for both sexes, it is notable that urban men in Antananarivo outspent all other residence, sex or region categories. There was no clear type of residence based trend in perceptions around fashion. While we expected that women and men in urban areas would value fashion more, we found that in Antananarivo peri-urban women were most likely to suggest they were often fashionable (19.7%); and peri-urban men were most likely to suggest they felt following fashion was important or very important (8%).

#### Region, residence and sexual behaviour

Reported sexual behaviour differed by region, although for men, unlike in previous studies, the contrast across region within urban areas was almost negligible, 58% in Antananarivo as compared to 61% in Antsiranana reported ever having had sex; and 33% versus 35% reported having multiple partners. For women, however, large differences remained. Only 42% of urban women in Antananarivo as compared to 62% of women in Antsiranana reported ever having had sex; and only 5.5% of urban Antananarivo women reported multiple partners as compared to 19.4% in Antsiranana. Similarly, women’s reports of having ever had sex with the intention of accessing money or materials in return were much higher in Antsiranana as compared to Antananarivo, although the percentages in our study were much lower than in previous studies, likely due to the change in the wording of the question. Regardless, a substantial difference remained with less than one percent of women reporting transactional sex in urban Antananarivo as compared to nearly 20% in urban Antsiranana.

Descriptive statistics provided only limited support to our hypothesis that the urban setting would be the riskier setting for sexual behaviour. For women, there appears to be a trend toward increased likelihood of reporting having had multiple sexual partners in the last year moving from only 1.8% in the rural areas to nearly 4% in the peri-urban zone and finally 5.5% for urban women. For men, this pattern was observed for having ever had sex, where far more young urban men reported having started sexual activity as compared to both their rural and peri-urban counterparts (58% as compared to 43% or 46% respectively). Apart from these findings, however, there was a stronger indication that within Antananarivo the peri-urban environment may be the riskier one for women, as the percentage of women who reported ever having had sex or ever having had transactional sex was highest in this zone of residence; the latter is particularly striking as nearly all of those who reported having practiced transactional sex resided in the peri-urban zone.

### Consuming modern goods and sexual behaviour

We ran a series of bivariate and descriptive statistics to explore *lamaody* characteristics (including consumption patterns, and lifestyle) by sexual behavioural outcomes (Table 4). The percentages in bold on Table 4 indicate where unadjusted logistic regression models were statistically significant.

There was some indication that perceptions concerning *lamaody* explained sexual behaviour for men; there was more evidence of this association at the bivariate level for women- particularly with respect to the extent to which women perceived they were fashionable. Both for multiple partners or transactional sex, women engaged in these activities were more likely to report that they were at least sometimes fashionable.

There were also associations with consumption or modern lifestyle characteristics. For example, the percentage of men or women who indicated they had obtained four or more *lamaody* items was higher for those who had reported multiple partners and the difference was statistically significant for men. Certain *lamaody* practices were only statistically significantly associated with having multiple partners, but not transactional sex, including cell phone and internet use, and alcohol consumption. For example, 21% of women who had had two or more partners, as compared to only 5% of women with only one partner, reported using alcohol in the last four weeks. The difference was not nearly as stark when comparing across those who had or had not reported engaging in transactional sex (10% versus 6.5%). Other characteristics were statistically significantly associated with both multiple partners and transactional sex including having gone to a nightclub (for both men and women) and having gotten to know a foreigner (for women). These findings lend support to the assertion that there is an association between interests in consumption or consumption practices and sexual behaviour.

### The role of ethnic origins and place of residence on sexual behaviour

In Table 5 we considered the association between ethnicity and sexual behaviour by zone of residence. This table only provides descriptive trends, we cannot account for every zone of residence nor test for significance within type of residence as the sample sizes become too small.

For men overall, a slightly lower percentage originating from *Imerina* (land of the Merina ethnic group) reported having had multiple partners as compared to those originating from outside of *Imerina* (28.5% versus 35.7%). The sharpest contrast was drawn in peri-urban Antananarivo, where 20.6% of those from *Imerina* report multiple partners as compared to 35% for those originating from other areas. Among men from *Imerina*, the highest percentage of those reporting multiple partners did not reside in urban Antananarivo, as expected, but rather in rural Antananarivo (33%).

Among women, overall the results are consistent with previous work examining sexual behaviour by province in Madagascar (which overlaps with ethnic groupings). The percentage of women who reported either having had multiple partners or having practiced transactional sex is lower among those who originated from *Imerina* as compared to those who originated from other areas. The results do not support our expectation that the urban zone fosters riskier sexual behaviour, at least not for women from *Imerina*. Rather, among women from *Imerina* who resided in urban Antananarivo, literally zero reported having had multiple partners or engaging in transactional sex. Conversely, women who originated from *Imerina* and resided in peri-urban Antananarivo were the most likely to report either sexual behaviour- 2.8% reported having had multiple partners in the last year and 5% report ever having engaged in transactional sex- a percentage marginally higher than that reported for those who originated from outside of *Imerina* and resided in peri-urban Antananarivo. Reports of multiple partners were low, yet still more prevalent in rural as compared to urban Antananarivo among women of *Imerina* origin. There is also evidence that current place of residence may influence sexual behaviour. While the percentage of women who reported having had multiple partners was similar for those who originated from outside of *Imerina* in both urban Antananarivo and urban Antsiranana (20.7% versus 19.6%); far fewer women from outside of *Imerina* reported having engaged in transactional sex in urban Antananarivo as compared to Antsiranana (2.7% as compared to 21.0%).^f^ In addition, among the small number of women who reported sexual exchange from any residence type in Antananarivo, 75% originated from *Imerina* (not shown). Taken together, findings suggest that both ethnicity and zone of residence influence sexual behaviour, but for zone of residence, not in the direction anticipated.

### Multivariate associations between consumption and sexual behaviour

Multivariate analysis provided the opportunity to assess the relative strength and association of type of residence, ethnic origin and modern lifestyle and consumption characteristics with sexual behavioural outcomes, controlling for socio-economics and socio-demographics (see Table 6). We caution the reader that the findings point to associations rather than causal relationships.

For men in the exploratory analyses, type of residence, perceptions around the importance of fashion, consuming alcohol, and obtaining fashionable goods were all significantly associated with reporting having had multiple partners in the last 12 months. In the full model presented in Table 6 only type of residence and having attended a nightclub remained highly statistically significant, and alcohol consumption was of borderline significance (p < .07). Among the socio-demographic and socio-economic controls, the model also suggests that the odds of having multiple partners decreased slightly for men who lived with their spouse as compared to a parent (p < .09); and increased significantly with personal expenditures. Overall, the strongest associations were personal income and having frequented a nightclub. The odds of having reported multiple partners were over three times higher for those men who report having spent at least 100,000 ariary (about 50 USD) in the previous month (n = 83 or almost 17% of men included in this model) as compared to those with very little or no income (p < .003); and over 2.2 times higher for those who reported recently frequenting a nightclub (p < .003). That peri-urban residents were the least likely to report multiple partners may be in part explained by the strong positive association between multiple partners and personal expenditures, as expenditures were lowest for peri-urban residents in Antananarivo. Together, the model suggests that men who had multiple partners were more likely to have engaged in a certain lifestyle (associated with going to nightclubs, and drinking alcohol) and had relatively high spending power.

The results for women lend themselves to a similar interpretation. Among women, multivariate analysis also suggested having multiple partners was associated with modern lifestyle characteristics; while interests in consuming fashion, although significant in bivariate and some exploratory analyses did not hold up in the multivariate analysis. As hypothesized, for women ethnic origin was highly significant; in fact, ethnic origin held the largest effect size and statistical significance of any covariate (p < .002). Among modern lifestyle characteristics, like men, having attended a nightclub highly increased the odds of reporting multiple partners; in addition, for women, so did having gotten to know a foreigner, having used the internet and having recently consumed alcohol. These may be gendered associations; focus groups suggested that women were relying increasingly on the internet to locate potential partners, many of whom were not residing in Madagascar (e.g. ‘foreigners’) and alcohol consumption was deemed less appropriate for women, but identified as *lamaody*. Among the socio-demographic and economic indicators, the odds of reporting multiple partners decreased with rising age and education levels; however, increased with household asset-based wealth. Despite some important differences, the models for both men and women pointed to an association with ‘modern’ lifestyle characteristics among relatively well-off youth, manifested for men through personal disposable income and for women through household-based asset wealth.

The multivariate models for transactional sex shared some similarities to those with multiple partners for women, and certainly there is some overlap in these populations- 39% of those women who reported having had multiple partners in the last 12 months also reported ever having had transactional sex. That said, there were important distinctions that may reflect different underlying motivations between having multiple partners and having engaged in transactional sex. In terms of our hypotheses, type of residence would have likely been significant, but because there were zero observations from rural Antananarivo, it was dropped from the analysis. Ethnic origin was again highly significant, indicating that the odds of having engaged in transactional sex were increased for women originating from outside of *Imerina* (p < .000). Unlike the model examining multiple partners, both modern lifestyle characteristics and interests and perceptions around fashion were significantly associated with transactional sex. Both having attended a nightclub and gotten to know a foreigner strongly increased the odds of reporting having engaged in transactional sex. Nightclubs serve as informal sex trade venues in many urban areas in Madagascar; and women who frequent such clubs to practice informal sex work in Antananarivo describe their objective as ‘looking for a foreign husband’ (see [60]). There were interesting inconsistencies in this model. For example, while perceiving oneself as at least sometimes fashionable significantly increased the odds of transactional sex, having consumed any fashionable goods in the last six months decreased the odds of transactional sex (with only borderline significance). In addition, unlike the model examining associations with multiple partners, household wealth was not significant, yet, receiving money from parents highly significantly decreased the odds of transactional sex (p < .000). There is indication therefore of both consumption of fashion, or an interest to do so, but also indication of financial constraint. Together, this lends some support to assertions that young women otherwise unable to access a modern lifestyle may rely on transactional sex as a means to this end. One additional analytic effort lends further limited support to such an assertion. We asked women about the kinds of gifts they received or items they purchased with money provided by their two most recent sexual partners. We categorized these gifts into “basic” (e.g. food, soap, medicines); “luxury” goods (clothing, jewellery, cell phones); or “both.” Among women who reported having ever had transactional sex, only 3.7% report receiving only basic goods, while among all other women in relationships, 20% report having received only basic goods.

## Discussion

Overall, the data presented here provide limited support for the community perception that interests in consumption are driving sexual behaviour among young people; the findings, however, also suggest that socio-cultural belief systems operating alongside place of residence are also significantly associated with sexual behaviour.

### Consumption and sexual behaviour

While bivariate analyses pointed to significant associations between sexual behaviour and interests in consumption of modern goods, most of these associations did not hold significance in multivariate models. The findings regarding multiple partnerships suggest that a certain lifestyle and ability to access it, both in the “imaginary” and in material, seem correlated with having multiple sexual partnerships- namely going to nightclubs and associating with foreigners. In addition, bivariate findings shown in Table 4 indicate that modern technology use was also associated with multiple partners, but not transactional sex; further supporting linkages between modern youth lifestyles and multiple partnerships for both men and women. Finally, the findings point to associations with spending power for both women and men. For men, those with the highest reported personal expenditures were more likely to have multiple partners; for women, those who came from non-poor households were more likely to report having had multiple partners. Taken together, this suggests a certain lifestyle, reserved perhaps for those who can afford to access it. This ‘modern’ youth lifestyle includes, as focus group participants who defined ‘*lamaody’* explained, going out to ‘party,’ having multiple partners, and keeping up with fashion. Young men with spending power, able to access modern lifestyle pursuits, were better positioned to attract partners who wished to do the same; while young women from non-poor households were more likely to take part in such a lifestyle. These findings suggest that women who report multiple partners are perhaps not necessarily engaging in sexual activity out of need, but out of choice. They also lend to the debates on the gendered economic power structures around sexual activity, as these data would suggest that women involved in these relationships may not be entering them from an inferior economic position.

Despite the fact that 39% of those women who reported having had multiple partners in the last 12 months had also reported having ever engaged in transactional sex, the models predicting these two sexual practices differed in important ways. The model predicting transactional sex does lend more support to the assertion made in focus group discussions and spoken on the streets that young women are “laying down their bodies for the love of *lamaody.*” In particular, it is interesting to note that the variable capturing whether or not the respondent had received pocket money from his/her parents only held significance in multivariate models for transactional sex. While it was of borderline significance and positively associated with reporting multiple partners for women (but not for men) in a bivariate model, it quickly lost significance in the presence of other covariates. This implies a motivation for engaging in transactional sex- in the absence of financial support from parents, young women seek support from partners. While this cross-sectional evidence is limited, that this motivation might be to consume *lamaody* is furthered by the association between the perception of being able to keep up with *lamaody* and transactional sex.

That said, the apparent interest in pursuing *lamaody* as suggested in these data may belie some more complex relationships. Cole (2004) describes how young women recognize the social importance of acquiring certain goods and attaining a certain look toward efforts at social mobility. Stoebenau similarly described women who “looked for foreign husbands” in nightclubs explaining that this venue required a certain kind of dress, without it, chances of successfully attracting a foreigner (or any man) were far less great [18]. Therefore, it could well be that women who frequent nightclubs and engage in transactional sex keep up with *lamaody* in order to pursue certain types of relationships (e.g. with foreigners), rather than engaging in transactional sex in order to keep up with *lamaody*.

### Zone of residence, ethnic origin and sexual behaviour

In Antananarivo, we did not find support for the assertion that large urban areas experiencing an influx of modern goods and global youth culture would most encourage risky sexual behaviour. Instead, it may be that the power of social sanctions against such practices in the urban center are strongly influencing sexual behaviour, or at the very least, the willingness to divulge such behaviour. Among the urban elite Merina, there is a very high premium placed on women’s chastity, while no such restriction applies to men. Therefore, there is little distinction in ethnic origin and sexual behaviour for men, while there is for women. Not a single woman who indicated she originated from *Imerina* reported multiple partners or transactional sex in urban Antananarivo. In addition to belief systems, the homogeneity of the ethnic composition of Antananarivo as compared to most other major urban centers in Madagascar may play a role. Drawing from “social disorganization thesis,” Benefo suggests there is a lower likelihood of informal regulation of sexual behaviour in urban environments where communities of migrants of different ethnic groups are juxtaposed, distancing people from those tight-knit communities that might regulate and report on their behaviour [67]. Bishai et al. found support for this thesis in Uganda where men residing in more ethnically diverse communities were more likely to engage in extramarital sex [68]. The city of Antananarivo is much less ethnically diverse than Antsiranana, and less diverse than one would expect of a national capital city [86]. The counter-argument is true in Antsiranana, which is an ethnically diverse city. In addition to greater ethnic diversity in Antsiranana, there are also a different set of assumptions and practices with regard to premarital sexual behaviour than those that have been operating for centuries. The strong social sanctioning of sexual behaviour in Antananarivo probably also explains the extent to which concerns about these practices reverberate through urban Merina communities. The “moral-panic” that has resulted generates the sense that these practices are more prevalent than they in fact seem to be. That said, surveillance studies of youth sexual behaviour in Madagascar have noted increases in reported transactional sex in both regions, albeit overall the rates being much lower in Antananarivo [56-58].

Examining the peri-urban and rural zones tells a different story, however, as the peri-urban zone emerges as one of more risky sexual behaviour; where among women transactional sex is more likely for those originating from *Imerina* as compared to other regions. This may be in part a reflection of the current economic realities in Madagascar, as the peri-urban region of Antananarivo is where the majority of Export Processing Zone jobs are located; a sector that was collapsing through the political crisis as it unraveled during data collection. In addition, peri-urban households are on the aggregate not as wealthy as urban households. The economic conditions during data collection may also explain the results in Antsiranana to some extent as well. Antsiranana was becoming increasingly dependent on tourism for its economy. Hotels and restaurants had multiplied rapidly in the first decade of the millennium. The political crisis had reduced the tourist population, and may have contributed to reported rates of exchanging sex *in order to* access money or material items. The findings suggest that perhaps exposure to goods and lifestyles is associated with transactional sex, particularly when it is coupled with uncertainty generating a sense of ‘economic vulnerability’ in environments such as peri-urban Antananarivo and Antsiranana.

#### Study limitations

This research effort was limited in at least three ways. First, the analysis is hampered by a relatively small sample size and low proportion of women, in particular, who reported either outcome behaviour (7.3% for multiple partners and 5.9% for transactional sex). We did not have enough power to detect difference in models including interaction terms, nor were multivariate region and sex stratified models robust. Therefore, we were not able to test the extent to which urban residence modified the relationship between consumption or lifestyle and sexual behaviour in our multivariate models, or examine in detail the extent to which associations between consumption and sex differed by region. Second, we are also limited by this being a cross-sectional study. We therefore cannot make any claims about the causal direction of these relationships, hindering the capacity to understand to what extent consumption *motivates* sexual behaviour. Third, and related, our independent and outcome variables are not sequenced appropriately to demonstrate causality within the context of a cross-sectional study design. We had to balance concerns with recall bias alongside our ability to more appropriately predict sexual behavioural outcomes. We used time frames that were consistent with other studies (where applicable), appropriate to the Madagascar context (going to an internet café may not be a frequent occurrence for some), and allowed for recall. Nonetheless, we stress that these models cannot make any causal claims. Our results do provide evidence of association in a new area of research examining the intersections between consumption, lifestyles and culture. Further research is warranted to capture a longitudinal understanding of the motivations for engaging in transactional sex or having multiple sexual partners.

## Conclusion

In this paper we provide one of the first known quantitative studies testing the association between the consumption of modern goods or lifestyle and sexual risk behaviour among youth in rural, peri-urban and urban Antananarivo as well as in urban Antsiranana. We expected levels of risky sexual behaviour would be associated with residing in more globalized urban centers; and would be associated with socio-cultural characteristics. We measured two outcomes: having had multiple partners in the last year; and having ever practiced transactional sex. We find that for both men and women, having multiple partnerships seems to be associated with characteristics of a global, youth lifestyle, marked by having frequented nightclubs, consumed alcohol, and in addition for women, used technology and met up with foreigners. We also find evidence of positive associations between wealth or expenditures and multiple partnerships for men and women; as well as very strong associations with ethnic origin for women, but not for men.

For transactional sex, our multivariate results do lend some support to the community assertions that women are exchanging sex in order to keep up with fashion: we found an association between perceptions of being able to keep up with fashion and transactional sex. In both descriptive and multivariate analyses the role of ethnic origin was very significant. The dominant culture in a region played a role in sexual outcomes and was a much more consistent and strong determinant of behaviour than interests in, or patterns of consumption.

Further research is necessary to understand the temporal relationships between consumption and sexual behaviour in order to better tease out to what extent an interest or a desire to consume motivates sexual behaviour; versus their being co-determined processes- youth who engage in what they perceive to be a more “modern” lifestyle adapt the habits (drinking, attending clubs, having multiple partners) of that lifestyle. In addition, research should account for the association between women’s rights and risky sexual behaviour; women in *Imerina* enjoy more rights and freedoms than women in many parts of Madagascar, and this may help to explain the regional distinctions in reliance on transactional sex.

Our findings suggest that socio-cultural belief systems must be emphasized when examining sexual behaviour and when trying to intervene toward the prevention of HIV transmission. In addition, our research points to the potential importance for hybrid spaces within the context of urbanization and globalization- the volatility in burgeoning peri-urban spaces may be of particular concern. Overall, while we find indication of an association between sexual behaviour and interest in modern goods, or modern lifestyles; such processes do not single-handedly explain risky sexual behaviour among youth, but they are shaped by culture and conditions of economic uncertainty.

## Endnotes

^a^The city of Antananarivo is located in what was officially recognized as the province of Antananarivo until 2007.

^b^While imports as a percent contribution of GDP were on average 6.8% higher than exports between 1960 and 1999, they have been on average 9.5% higher than exports since 2000 [45].

^c^In Malagasy: “Efa nisy fotoana ve ianao nanao firaisana ara-nofo tamin’ny olona ka niandrasanao fanomezana, na vola na zavatra ho takalon’izany?”

^d^While this is a far better approximation of ethnic origin than is typically available, it still has limitations. Mixed-ethnic marriages are far from negligible, and respondents may not primarily identify with the ethnic group which resides in the region indicated by their father’s tomb. That said, most ethnic groups in Madagascar follow patrilineal and patrilocal kinship and residence patterns.

^e^The MCA included the following variables: number of rooms, type of wall, floor, ceiling and roof; energy for cooking and for lighting; type of toilet; source for water; ownership of land for cultivation; number of tables, chairs, living room sets, cars, motorcycles, refrigerators, cattle, television and cell phones. While meant to capture a respondent’s background socio-economic status, the household based asset index is not ideal as it is based on the respondent’s current household, which may differ from their natal household.

^f^This comparison should be made with caution for a number of reasons, primarily because the composition of ethnic groups (other than Merina) residing in Antananarivo does not correspond to the composition in Antsiranana; and the sample sizes are much smaller in urban Antananarivo among those who do not originate from *Imerina*.

## Competing interests

None of the authors have competing interests to declare.

## Authors’ contributions

KS led the conception of the project, the study design, analysis and wrote the majority of the paper. RN contributed to the study design, sampling frame, provided oversight on the analytic approaches and findings, and contributed to data presentation and writing of the results. VR led the quantitative data collection, the design of the study instrument, writing of the study methodology, and significantly contributed to the analysis. PGR and VR led the qualitative data collection, contributed to the design of the survey instrument and to quantitative data collection, and with KS analysed the qualitative data. RL helped conceive of the analysis, contributed to data interpretation, and helped draft sections of the manuscript. All authors read and approved the final manuscript.

## References

[B1] Selikow T-A Bheki Z Cedras E The Ingagara, the Regte and the Cherry: HIV/AIDS and Youth Culture in Contemporary Urban Townships Agenda 2002 53 22 32

[B2] Leclerc-Madlala S Transactional sex and the pursuit of modernity Social Dynamics-A Journal of the Centre for African Studies University of Cape Town 2003 29 213 233

[B3] Cole J Fresh contact in Tamatave, Madagascar: sex, money, and intergenerational transformation American Ethnologist 2004 31 573 588 10.1525/ae.2004.31.4.573

[B4] Halperin DT Epstein H Concurrent sexual partnerships help to explain Africa’s high HIV prevalence: implications for prevention Lancet 2004 364 4 6 15234834 10.1016/S0140-6736(04)16606-3

[B5] Epstein H Morris M Concurrent partnerships and HIV: an inconvenient truth J Int AIDS Soc 2011 14 13 10.1186/1758-2652-14-13 21406080 PMC3064618

[B6] Mah TL Shelton JD Concurrency revisited: increasing and compelling epidemiological evidence J Int AIDS Soc 2011 14 33 10.1186/1758-2652-14-33 21689437 PMC3133533

[B7] Dunkle KL Jewkes RK Brown HC Gray GE McIntryre JA Harlow SD Transactional sex among women in Soweto, South Africa: prevalence, risk factors and association with HIV infection Soc Sci Med 2004 59 1581 1592 10.1016/j.socscimed.2004.02.003 15279917

[B8] Maganja RK Maman S Groves A Mbwambo JK Skinning the goat and pulling the load: transactional sex among youth in Dar es Salaam, Tanzania AIDS Care 2007 19 974 981 10.1080/09540120701294286 17851993

[B9] Wojcicki JM Commercial sex work or ukuphanda? Sex-for-money exchange in Soweto and Hammanskraal area, South Africa Cult Med Psychiatry 2002 26 339 370 10.1023/A:1021291922026 12555904

[B10] Wardlow H Anger, economy, and female agency: problematizing “prostitution” and “sex work” among the Huli of Papua New Guinea Signs 2004 29 1017 1040 10.1086/382628

[B11] Fordham G Moral panic and the construction of national order - HIV/AIDS risk groups and moral boundaries in the creation of modern Thailand Critique of Anthropology 2001 21 259 316 10.1177/0308275X0102100301

[B12] Hunter M The materiality of everyday sex: thinking beyond ‘prostitution’ African Studies 2002 61 99 120 10.1080/00020180220140091

[B13] Wojcicki JM “She drank his money”: survival sex and the problem of violence in taverns in Gauteng Province, South Africa Medical Anthropology Quarterly 2002 16 267 293 10.1525/maq.2002.16.3.267 12227257

[B14] Luke N Kurz KM Cross-generational and Transactional Sexual Relations in Sub-Saharan Africa: Prevalence of Behavior and Implications for Negotiating Safer Sexual Practices 2002 Washington, D.C: AIDSMark, USAID Project

[B15] Kuate-Defo B Young people’s relationships with sugar daddies and sugar mummies: what do we know and what do we need to know? Afr J Reprod Health 2004 8 13 37 10.2307/3583175 15623116

[B16] Andriamahenina R Tsaboto J Projet multi-sectoriel de lutte contre le VIH/SIDA (PMLS) Les us et coutumes Malagasy lies aux relations sexeulles 2002 Antananarivo: The World Bank

[B17] Nshindano C Maharaj P Reasons for multiple sexual partnerships: perspectives of young people in Zambia African Journal of AIDS Research 2008 7 37 44 10.2989/AJAR.2008.7.1.5.433 25871270

[B18] Stoebenau K From those who “have to carry men” to those who “look to marry men”: The social organization of women’s sex work in Antananarivo, Madagascar. Dissertation 2006 Johns Hopkins University

[B19] Stoebenau K Nixon SA Rubincam C Willan S Zembe YZN Tsikoane T More than just talk: the framing of transactional sex and its implications for vulnerability to HIV in Lesotho, Madagascar and South Africa Globalization and Health 2011 7 34 10.1186/1744-8603-7-34 21961516 PMC3204228

[B20] Kaufman CE Stavrou SE ‘Bus fare please’: the economics of sex and gifts among young people in urban South Africa Cult Health Sex 2004 6 377 391 10.1080/13691050410001680492

[B21] Smith DJ Modern marriage, men’s extramarital sex, and HIV risk in southeastern Nigeria Am J Public Health 2007 97 997 1005 10.2105/AJPH.2006.088583 17463366 PMC1874209

[B22] Leclerc-Madlala S Age-disparate and intergenerational sex in southern Africa: the dynamics of hypervulnerability AIDS 2008 22 S17 S25 19033752 10.1097/01.aids.0000341774.86500.53

[B23] Preston-Whyte E Varga C Oosthuizen H Roberts R Blose F Parker R, Barbosa RM, Aggleton P Framing the Sexual Subject: The Politics of Gender, Sexuality, and Power 2000 Berkeley, CA: University of California Press 165 191

[B24] Mill JE Anarfi JK HIV risk environment for Ghanaian women: challenges to prevention Soc Sci Med 2002 54 325 337 10.1016/S0277-9536(01)00031-4 11824910

[B25] De Vogli R Birbeck GL Potential impact of adjustment policies on vulnerability of women and children to HIV/AIDS in Sub-Saharan Africa J Health Popul Nutr 2005 23 105 120 16117362

[B26] Schoepf BG Women, aids, and economic-crisis in Central-Africa Canadian Journal of African Studies-Revue Canadienne des Etudes Africaines 1988 22 625 644

[B27] Setel PW A Plague of Paradoxes: AIDS, culture, and Demography in Northern Tanzania 1999 Chicago: The University of Chicago Press

[B28] Dilger H Sexuality, AIDS, and the lures of modernity: reflexivity and morality among young people in rural Tanzania Medical Anthropology: Cross Cultural Studies in Health and Illness 2003 22 23 52 10.1080/01459740306768 12641295

[B29] Luke N Confronting the ‘sugar daddy’ stereotype: Age and economic asymmetries and risky sexual behavior in urban Kenya Int Fam Plan Perspect 2005 31 6 14 10.1363/3100605 15888404

[B30] Smith DJ “These girls today na war-o”: premarital sexuality and modern identity in southeastern Nigeria Africa Today 2001 47 98 120

[B31] Hawkins K Price N Mussa F Milking the cow: young women’s construction of identity and risk in age-disparate transactional sexual relationships in Maputo, Mozambique Glob Public Health 2009 4 169 182 10.1080/17441690701589813 19333807

[B32] Silberschmidt M Rasch V Adolescent girls, illegal abortions and “sugar-daddies” in Dar es Salaam: vulnerable victims and active social agents Soc Sci Med 2001 52 1815 1826 10.1016/S0277-9536(00)00299-9 11352408

[B33] Ramamonjisoa J Robinson S Sociologie et Sida Cahier des Sciences Sociales, revue du département de sociologie, Université d’Antananarivo in press

[B34] Wamoyi J Fenwick A Urassa M Zaba B Stones W “Women’s Bodies are Shops”: beliefs about transactional sex and implications for understanding gender power and HIV prevention in Tanzania Arch Sex Behav 2011 40 5 15 10.1007/s10508-010-9646-8 20652390

[B35] Luke N Economie status, informal exchange, and sexual risk in Kisumu, Kenya Economic Development and Cultural Change 2008 56 375 396 10.1086/522896 PMC4297648 25605976

[B36] Luke N Exchange and condom use in informal sexual relationships in urban Kenya Economic Development and Cultural Change 2006 54 319 348 10.1086/497011

[B37] Bene C Merten S Women and fish-for-sex: transactional sex, HIV/AIDS and gender in African fisheries World Dev 2008 36 875 899 10.1016/j.worlddev.2007.05.010

[B38] Cole J The Jaombilo of Tamatave (Madagascar), 1992–2004: reflections on youth and globalization Journal of Social History 2005 38 891 914 10.1353/jsh.2005.0051

[B39] Masvawure T ‘I just need to be flashy on campus’: female students and transactional sex at a university in Zimbabwe Cult Health Sex 2010 12 857 870 10.1080/13691050903471441 20069476

[B40] Covell M Historical Dictionary of Madagascar 1995 London: The Scarecrow Press, Inc.

[B41] Glick P Roubaud F Export processing zone expansion in Madagascar: what are the labour market and gender impacts? Journal of African Economies 2006 15 722 756 10.1093/jae/ejk016

[B42] Nicita A Razzaz S Who Benefits and How Much? How Gender Affects Welfare Impacts of a Booming Textile Industry. 3029 2003 Washington, D.C: The World Bank, Trade, Development Research Group and Gender Division, Poverty Reduction and Economic Managment Network Policy Research Working Paper

[B43] Kapur D Webb R Governance-related Conditionalities of the International Financial Institutions. 6, 1–28 2000 New York: United Nations Conference on Trade and Development G-24 Discussion Paper No. 6

[B44] Kovach H Lansman Y World Bank and IMF conditionality: a development injustice 2006 Eurodad 1 33

[B45] The World Bank The World Bank Group: World Development Indicators Online 2008 The World Bank (http://data.worldbank.org/data-catalog/world-development-indicators)

[B46] Directeur Régional de l’Environnement des Forêts et du Tourisme Personal Communication with Office of Direction Régionale de l’Environnement des forêts et du Tourisme, Antsiranana 200923388578

[B47] Antoine P Razafindrakoto M Roubaud F **Contraints de rester jeunes ? Evolution de l'insertion urbaine dans trois capitales africaines: Dakar, Yaoundé, Antananarivo** Les jeunes, hantise de l'espace public dans les sociétés du Sud 2001 Paris: IRD-Editions/Editions de l'Aube

[B48] Zegers M Synthesis Report: Society, Culture and HIV/AIDS in Madagascar 2003 The World Bank: Antananarivo 1 98

[B49] Rakotomalala MM Contribution à une anthropologie de la sexualité Merina (Centre de Madagascar). Dissertation 1996 Paris: Institut National des langues et civilisations oreintales

[B50] Ramilison EN La prostitution chez les mineures et les jeunes de trois grandes villes malgaches: Est-ce que un travail ou un style de vie? 2004 Antananarivo: INSTAT

[B51] Huntington R Gender and Social Structure in Madagascar 1988 Indiana: Indiana University Press

[B52] Feeley-Harnik G A Green Estate: Restoring Independence in Madagascar 1991 Washington, D.C.: Smithsonian Institution Press

[B53] INSTAT, Macro International Inc Enquête Démographique et de Santé, Madagascar 1997 1998 Calverton, MD U.S.A: INSTAT; Macro International Inc

[B54] INSTAT, ORC Macro Enquête Démographique et de Santé de Madagascar 2003–2004 2005 Calverton, MD U.S.A: INSTAT; ORC Macro

[B55] INSTAT, Projet MADIO L'emploi, le chômage et les conditions d'activité des ménages dans les sept grandes villes de Madagascar. 1–76 2001 Antananarivo: INSTAT, Projet MADIO

[B56] INSTAT, Comité National de la Lutte contre le SIDA Enqûete de Surveillance Comportementale à Madagascar--Jeunes--2004 2004 Antananarivo: INSTAT, Comité National de la Lutte contre le SIDA

[B57] INSTAT, Comité National de la Lutte contre le SIDA CNLS Enquête de Surveillance Comportementale à Madagascar--Jeunes--2006 2007 Antananarivo: CNLS

[B58] INSTAT, Comité National de la Lutte contre le SIDA CNLS Enquête de Surveillance Comportementale à Madagascar--Jeunes--2008 2009 Antananarivo: CNLS

[B59] Mangalaza E Sous la mousticaire: fidélité conjugale et libertinage chez les Betsimisaraka: L’amour Cahiers-ethnologiques 1993 15 61 71

[B60] Stoebenau K Symbolic capital and health: the case of women’s sex work in Antananarivo, Madagascar Soc Sci Med 2009 68 2045 2052 10.1016/j.socscimed.2009.03.018 19362403

[B61] Ramamonjisoa J Personal Communication with Author, Sociologist-Anthropologist Janine Ramamonjisoa in Antananarivo 2003

[B62] Rakotomalala MMe-mail correspondence with the author- Malanjaona Manoelina Rakotomalala. 2005 2005

[B63] Comité National de lutte contre le VIH/SIDA, Ministère de la Santé et du Planning Familial Résultats Préliminaires de l’Enquête de Surveillance Biologique du VIH/SIDA et de la Syphilis, Année 2004–2005. Edited by CNLS. 1–20 2005 Antananarivo: CNLS

[B64] Comité National de lutte contre le VIH/SIDA, Ministère de la Santé et du Planning Familial CNLS Résultats Préliminaires de l’Enquête de Surveillance Biologique du VIH/SIDA et de la Syphilis, Année 2007 2008 Antananarivo: CNLS

[B65] Potts M Halperin DT Kirby D Swidler A Marseille E Klausner JD Public health: reassessing HIV prevention Science 2008 320 749 750 10.1126/science.1153843 18467575 PMC3501984

[B66] Green EC Mah TL Ruark A Hearst N A framework of sexual partnerships: risks and implications for HIV prevention in Africa Stud Fam Plann 2009 40 63 70 10.1111/j.1728-4465.2009.00187.x 19397186

[B67] Benefo KD Determinants of Zambian men’s extra-marital sex: a multi-level analysis Arch Sex Behav 2008 37 517 529 10.1007/s10508-007-9243-7 17999170

[B68] Bishai D Patil P Pariyo G Hill K The babel effect: community linguistic diversity and extramarital sex in Uganda AIDS Behav 2006 10 369 376 10.1007/s10461-006-9097-3 16604296

[B69] Romero-Daza N Multiple sexual partners, migrant labor, and the makings for an epidemic: knowledge and beliefs about AIDS among women in highland Lesotho Hum Organ 1994 53 192 205

[B70] White L The Comforts of Home: Prostitution in Colonial Nairobi 1990 Chicago: The University of Chicago Press

[B71] Norris AH Kitali AJ Worby E Alcohol and transactional sex: how risky is the mix? Soc Sci Med 2009 69 1167 1176 10.1016/j.socscimed.2009.07.015 19713023

[B72] Hallman K Gendered socioeconomic conditions and HIV risk behaviours among young people in South Africa African Journal of AIDS Research 2005 4 37 50 10.2989/16085900509490340 25865640

[B73] Robinson J Yeh E Transactional sex as a response to risk in Western Kenya American Economic Journal-Applied Economics 2011 3 35 64

[B74] Dunkle KL Wingood GM Camp CM DiClemente RJ Economically motivated relationships and transactional sex among unmarried African American and white women: results from a US National Telephone Survey Public Health Rep 2010 125 90 100 20626196 10.1177/00333549101250S413 PMC2882979

[B75] Addai I Ethnicity and sexual behavior in Ghana Soc Biol 1999 46 17 32 10842499 10.1080/19485565.1999.9988985

[B76] Li SH Huang H Cai Y Xu G Huang FR Shen XM Characteristics and determinants of sexual behavior among adolescents of migrant workers in Shangai (China) BMC Public Health 2009 9 195 10.1186/1471-2458-9-195 19538756 PMC2706248

[B77] Hallman KK Researching the Determinants of Vulnerability to HIV among Adolescents Ids Bulletin-Institute of Development Studies 2008 39 36

[B78] Bingenheimer JB Men’s multiple sexual partnerships in 15 sub-Saharan African countries: Sociodemographic patterns and implications Stud Fam Plann 2010 41 1 17 10.1111/j.1728-4465.2010.00220.x 21151707 PMC2998893

[B79] Dunkle KL Jewkes R Nduna M Jama N Levin J Sikweyiya Y Transactional sex with casual and main partners among young South African men in the rural Eastern Cape: prevalence, predictors, and associations with gender-based violence Soc Sci Med 2007 65 1235 1248 10.1016/j.socscimed.2007.04.029 17560702 PMC2709788

[B80] Booysen F Van der Berg S Burger R Von Maltitz M du Rand G Using an asset index to assess trends in poverty in seven Sub-Saharan African countries World Dev 2008 36 1113 1130 10.1016/j.worlddev.2007.10.008

[B81] Dimbuene ZT Defo BK Family environment and premarital intercourse in bandjoun (West Cameroon) Arch Sex Behav 2012 41 351 361 10.1007/s10508-011-9830-5 21904944

[B82] Smith D Roofe M Ehiri J Campbell-Forrester S Jolly C Jolly P Sociocultural contexts of adolescent sexual behavior in rural Hanover, Jamaica J Adolesc Health 2003 33 41 48 10.1016/S1054-139X(02)00563-3 12834996

[B83] Do M Meekers D Multiple sex partners and perceived risk of HIV infection in Zambia: attitudinal determinants and gender differences Aids Care-Psychological and Socio-Medical Aspects of Aids/Hiv 2009 21 1211 1221 10.1080/09540120902730047 20024696

[B84] Harrison A Cleland J Frohlich J Young People’s Sexual Partnerships in KwaZulu-Natal, South Africa: patterns, Contextual Influences, and HIV Risk Stud Fam Plann 2008 39 295 308 10.1111/j.1728-4465.2008.00176.x 19248716 PMC3848499

[B85] Kempadoo K Freelancers, temporary wives, and beach-boys: Researching sex work in the Caribbean Feminist Review 2001 39 62

[B86] Roubaud F Identités et Transition Démocratique: L’exception Malgache? 2000 Paris: L’Harmattan

